# Medicines of the Shetlanders

**Published:** 1860-07

**Authors:** 


*Medicines of the Shetlanders.*—Their popular receipts are scurvy-grass for cutaneous complaints, butter-milk for dropsy, shells of whelks calcined and pounded for dyspepsia, and a variety of steatite for excoriations.

